# The ancestral shape hypothesis: an evolutionary explanation for the occurrence of intervertebral disc herniation in humans

**DOI:** 10.1186/s12862-015-0336-y

**Published:** 2015-04-27

**Authors:** Kimberly A Plomp, Una Strand Viðarsdóttir, Darlene A Weston, Keith Dobney, Mark Collard

**Affiliations:** Human Evolutionary Studies Program and Department of Archaeology, Simon Fraser University, Burnaby, BC, Canada; Biomedical Center, University of Iceland, Reykjavik, Iceland; Department of Anthropology, University of British Columbia, Vancouver, BC, Canada; Department of Human Evolution, Max Planck Institute for Evolutionary Anthropology, Leipzig, Germany; Department of Archaeology, University of Aberdeen, Aberdeen, UK

**Keywords:** Back pain, Disc herniation, Vertebral shape, Bipedalism, Geometric morphometrics, Schmorl’s nodes

## Abstract

**Background:**

Recent studies suggest there is a relationship between intervertebral disc herniation and vertebral shape. The nature of this relationship is unclear, however. Humans are more commonly afflicted with spinal disease than are non-human primates and one suggested explanation for this is the stress placed on the spine by bipedalism. With this in mind, we carried out a study of human, chimpanzee, and orangutan vertebrae to examine the links between vertebral shape, locomotion, and Schmorl’s nodes, which are bony indicators of vertical intervertebral disc herniation. We tested the hypothesis that vertical disc herniation preferentially affects individuals with vertebrae that are towards the ancestral end of the range of shape variation within *Homo sapiens* and therefore are less well adapted for bipedalism.

**Results:**

The study employed geometric morphometric techniques. Two-dimensional landmarks were used to capture the shapes of the superior aspect of the body and posterior elements of the last thoracic and first lumbar vertebrae of chimpanzees, orangutans, and humans with and without Schmorl’s nodes. These data were subjected to multivariate statistical analyses.

Canonical Variates Analysis indicated that the last thoracic and first lumbar vertebrae of healthy humans, chimpanzees, and orangutans can be distinguished from each other (p<0.028), but vertebrae of pathological humans and chimpanzees cannot (p>0.4590). The Procrustes distance between pathological humans and chimpanzees was found to be smaller than the one between pathological and healthy humans. This was the case for both vertebrae. Pair-wise MANOVAs of Principal Component scores for both the thoracic and lumbar vertebrae found significant differences between all pairs of taxa (p<0.029), except pathological humans *vs* chimpanzees (p>0.367). Together, these results suggest that human vertebrae with Schmorl’s nodes are closer in shape to chimpanzee vertebrae than are healthy human vertebrae.

**Conclusions:**

The results support the hypothesis that intervertebral disc herniation preferentially affects individuals with vertebrae that are towards the ancestral end of the range of shape variation within *H. sapiens* and therefore are less well adapted for bipedalism. This finding not only has clinical implications but also illustrates the benefits of bringing the tools of evolutionary biology to bear on problems in medicine and public health.

**Electronic supplementary material:**

The online version of this article (doi:10.1186/s12862-015-0336-y) contains supplementary material, which is available to authorized users.

## Background

Back pain is an important health issue. It has been estimated that 22-65% of people will experience back pain at some point in their lives [1], making it one of the most common health problems [2]. Back pain is also one of the most serious health problems. Recent work suggests that it is the greatest contributor to disability on a global scale [3]. The prevalence of back pain and the frequency with which it causes disability mean that it can impose a substantial economic burden on countries [4]. For example, the annual cost of back pain in the UK has been estimated to exceed £1.5 billion per year [5]. Given the importance of back pain, there is a need for greater understanding of the underlying factors that cause it.

Intervertebral disc herniation is a widespread but poorly understood cause of back pain [6]. It is defined as a prolapse of the gelatinous substance inside the disc, the nucleus pulposus, either horizontally through the fibrous outer disc layers or vertically into the vertebral endplate [6]. Intervertebral disc herniation is frequent among adults, with recent studies suggesting that prevalence rates range from 20% to 78%, depending on population [7-9]. Numerous potential causes of intervertebral disc herniation have been proposed, including genetic predisposition, disc composition, developmental issues, and physical strain or trauma [10-15], but the aetiology and pathogenesis of the condition remain unclear [16].

Recently, a number of studies have suggested that vertebral shape may affect the propensity to experience intervertebral disc herniation. Pfirrmann and Resnick [17] found that Schmorl’s nodes were associated with a flat vertebral endplate as opposed to the more common concave endplate in a sample of cadavers. Schmorl’s nodes are depressions on the upper and lower surfaces of the vertebral body that result from vertical intervertebral disc herniation [18]. They can be identified with the use of medical imaging technology [19,20] or on dry bone [21-23]. Harrington et al. [24] obtained similar results to Pfirrmann and Resnick [23]. They found that the size and shape of the vertebral body was associated with lower lumbar intervertebral disc herniation in a large sample of clinical patients. Most recently, Plomp et al. [25] found a correlation between lower thoracic vertebral shape and the presence of Schmorl’s nodes in Medieval and Post-Medieval skeletons. They concluded that the shape of the pedicles and vertebral body might play a role in the development of Schmorl’s nodes [25].

Given that several studies have suggested a link between vertebral shape and the propensity to experience intervertebral disc herniation, there is reason to investigate possible explanations for why certain vertebral shapes should predispose for this condition. Humans display substantially more degenerative and traumatic spinal pathologies than non-human primates [26,27]. This has led some researchers to hypothesize that our unique mode of locomotion, bipedalism, may influence the development of these conditions [28-30]. With this theory in mind, we carried out a cross-species study of vertebral shape variation in humans and non-human apes to examine the links between vertebral shape, locomotor behaviour and vertical intervertebral disc herniation. Specifically, we tested the hypothesis that intervertebral disc herniation preferentially affects individuals whose vertebral shape are towards the ancestral end of the range of shape variation within *Homo sapiens* and therefore are less well adapted for bipedalism.

This “ancestral shape hypothesis” is derived from work on the evolution of bipedalism. It is now generally accepted that humans and other hominins are more closely related to chimpanzees (*Pan troglodytes*), and bonobos (*Pan paniscus*) than they are to any other living species [31]. At the moment, the locomotor behaviour of the common ancestor of the hominin and chimpanzee/bonobo lineages is debated. A number of different locomotor behaviours have been suggested to be antecedent to bipedalism [32-34]. The most frequently cited suggestion is that the common ancestor was a knuckle-walker like chimpanzees, bonobos, and gorillas (*Gorilla gorilla*) [35]. However, it has also been argued that the common ancestor of the hominin and chimpanzee/bonobo lineages was an arboreal quadrumanous climber like orangutans (*Pongo pygmaeus*) [36]. Depending on which of these hypotheses is correct, the hominin lineage shifted from knuckle-walking to bipedalism or from quadrumanous climbing to bipedalism. In both cases, the demands placed on the vertebrae would have changed. Selection likely acted to improve the ability of the vertebrae to cope with the new demands, but given that vertebral shape is almost certainly influenced by multiple genes and that the spine is multifunctional, we can also expect that within a hominin species, some individuals will have vertebrae that are closer in shape to those of the common ancestor than others. Given that the ancestral vertebral shape would not have been adapted for bipedalism, individuals whose vertebrae are towards the ancestral end of the range of shape variation can be expected to suffer disproportionately from external load-related spinal pathologies.

In our study, we employed geometric morphometrics (GM) to record and analyze vertebral shape. Being based on coordinate data as opposed to the inter-landmark distances of standard morphometrics, GM methods allow patterns of shape variation to be investigated within a well-understood statistical framework that yields easily interpreted numerical and visual results [37-40]. To identify vertebral shapes associated with bipedalism, we adopted the approach employed by Russo [41] and compared human vertebrae to the vertebrae of a knuckle-walker (the chimpanzee) and the vertebrae of a quadrumanous climber (the orangutan). Humans, chimpanzees, and orangutans vary in modal vertebral formulae, with 12 thoracic and 5 lumbar vertebrae in humans, 12 thoracic and 4 lumbar vertebrae in orangutans, and 13 thoracic and 3 to 4 lumbar vertebrae in chimpanzees [42]. Consequently, the last thoracic (T12/13) and the first lumbar (L1) vertebrae were included in the study to ensure positional homology between vertebrae of different species and to represent the functionally distinct thoracic and lumbar spines. Another important consideration was that human T12s and L1s are commonly afflicted by Schmorl’s nodes [20] and previous studies have found their shapes to correlate with the presence of these lesions [25,43].

Following Plomp et al. [25], the presence of Schmorl’s nodes was used as an indicator of vertical intervertebral disc herniation. We tested two predictions of the ancestral shape hypothesis: 1) there should be differences in shape between healthy human, chimpanzee, and orangutan vertebrae; and 2) human vertebrae with evidence of vertical intervertebral disc herniation should be more similar in shape to the vertebrae of chimpanzees or orangutans than are human vertebrae without evidence for intervertebral disc herniation.

## Methods

Last thoracic and first lumbar vertebrae from 71 humans, 36 chimpanzees, and 15 orangutans were included in the sample (Table 1). Only adult individuals were included in the analysis. Due to preservation issues and curation practices, not all individuals had both vertebrae present. In total, the sample comprised 114 human vertebrae (59 thoracic, 55 lumbar), 56 chimpanzee vertebrae (25 thoracic, 31 lumbar), and 27 orangutan vertebrae (12 thoracic, 15 lumbar). The human vertebrae analysed in this study are the same as those analysed by Plomp et al. [43]. They are Medieval-period specimens from the sites of Fishergate House, York [44], and Coach Lane, North Shields [45], and are curated at Durham University, UK (see Additional file 1 for details). Of the 114 human vertebrae, 54 exhibited Schmorl’s nodes, and 60 did not. For the purposes of this paper, we will refer to the former as “pathological” and the latter as “healthy”. The chimpanzee and orangutan vertebrae are housed at the American Museum of Natural History, New York, and the Smithsonian National Museum of Natural History, Washington DC, and are a mixture of zoo and wild-caught animals. None of the non-human ape vertebrae exhibit signs of pathology.

**Table 1:** **Composition of sample of vertebrae from the 71 humans, 36 chimpanzees, and 15 orangutans included in study**

**Taxon**	**Female**	**Male**	**Unknown**	**Total**
Orangutans				
Last thoracic	4	8	0	**12**
First lumbar	5	9	1	**15**
Combined	9	17	1	**27**
Chimpanzees				
Last thoracic	8	17	0	**25**
First lumbar	6	19	6	**31**
Combined	14	36	6	**56**
Healthy humans				
Last thoracic	12	14	0	**26**
First lumbar	15	17	2	**34**
Combined	27	31	2	**60**
Pathological humans				
Last thoracic	13	20	0	**33**
First lumbar	6	13	2	**21**
Combined	19	33	2	**54**

Pathological humans = human vertebrae with Schmorl’s nodes. Healthy humans = human vertebrae without Schmorl’s nodes or other pathologies. None of the orangutan or chimpanzee vertebrae were pathological.

**Figure 1:**
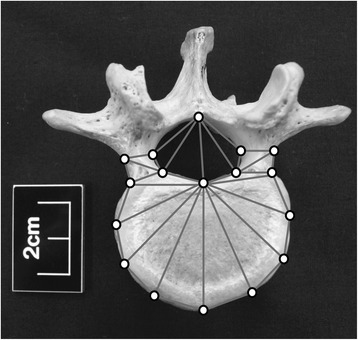
**Location of the 17 landmarks used to capture the superior aspect of vertebrae.** The eight landmarks on the posterior elements are Type II and the nine landmarks along the curve of the body are semi-landmarks. The vertebra depicted here is a human L1.

The dataset comprised the 2D Cartesian coordinates of 17 landmarks recorded on 197 dry-bone vertebrae (Figure 1). The landmarks were based on those used by Plomp et al. [25]. As we explained earlier, these authors found an association between certain vertebral shapes and the presence of Schmorl’s nodes in humans. The landmarks capture the outline shape of the pedicles, the neural foramen, and the superior aspect of the vertebral body [25]. Eight are Type II landmarks; the remainder are semi-landmarks [46]. The landmarks were recorded on standardized digital photographs with the aid of TPS Dig [47].

The first step in a geometric morphometric analysis is to reduce the effects of confounding factors [38]. As vertebrae are symmetrical along the sagittal midline, we followed the protocol outlined by Klingenberg et al. [48] to remove the influence of asymmetry on the results. To begin with, we created two datasets, one comprising the original landmark coordinates and the other the reflected and relabelled landmark coordinates [49]. We then slid the semi-landmarks to remove shape differences arising from the small differences that occur in the placement of semi-landmarks [50,51]. Next, we subjected the coordinates of the Type II landmarks and slid semi-landmarks of both datasets to generalized Procrustes analysis (GPA) [50,51]. GPA is designed to remove translation, rotational, and size effects [38]. Lastly, asymmetry was removed by calculating the average Procrustes coordinates between the original and reflected landmarks. These coordinates were used in all further analyses. The reflection, sliding procedure, and GPA were applied separately to the T12/T13 and L1 vertebrae. The semi-landmarks were slid, and the GPA performed, with the aid of TPSRelW [47].

Intra observer error was assessed as per Neubauer et al. [52]. A T12 vertebra and an L1 vertebra were each digitized ten times, and the greatest Procrustes distance between the repeated measurements for a given specimen was then compared to the smallest Procrustes distance among all specimens of the same type. In both analyses, the between-specimen distances were close to three times greater than the within-specimen distances. According to Neubauer et al. [52], this level of difference indicates that intra-observer error is unlikely to be a confounding factor. This analysis was carried out with Morphologika [53].

The impact of allometry was assessed by regressing the Procrustes coordinates on log centroid size. The statistical significance of male–female shape differences was determined using MANOVAs on all principal component (PC) scores obtained through principal components analyses (PCA). These analyses were performed in SPSS 16.0 [54] and MorphoJ [55], and carried out separately for the last thoracic and first lumbar vertebrae. Allometry was found to be a factor in vertebral shape (T12/T13: r^2^ = 0.092, p < 0.001; L1: r^2^ = 0.072, p < 0.001), but sexual dimorphism was not (p > 0.10). The frequency of Schmorl’s nodes between the two human populations was not statistically different (χ^2^ p > 0.339) and there was no statistical difference in vertebral shape between human populations (p > 0.108). In light of these results, we opted to employ allometry-free regression residuals derived from pooled-sex samples in the remainder of the analyses [56], with humans analyzed as a homogeneous population.

Following Klingenberg and Monteiro [57], we applied canonical variates analysis (CVA) to the pooled-sex regression residuals to determine the maximum Procrustes distances among taxa. The significance of differences was assessed using permutations of pair-wise Procrustes distances among all possible pairs of taxa. We carried this out initially for the last thoracic vertebrae and repeated it for the first lumbar vertebrae. The analyses were conducted in MorphoJ [55].

We used PCA to explore the pattern of inter-taxon shape variation [38]. Only PCs representing at least 5% of the total variance were considered in order to minimize noise from higher components [58]. The statistical significance of inter-taxon PCA score differences was assessed using MANOVAs. As in the previous analysis, the last thoracic and first lumbar vertebrae were analyzed separately. This analysis was performed in TPSRelW [47] and SPSS 16.0 [54].

## Results

### Last thoracic vertebrae

**Figure 2:**
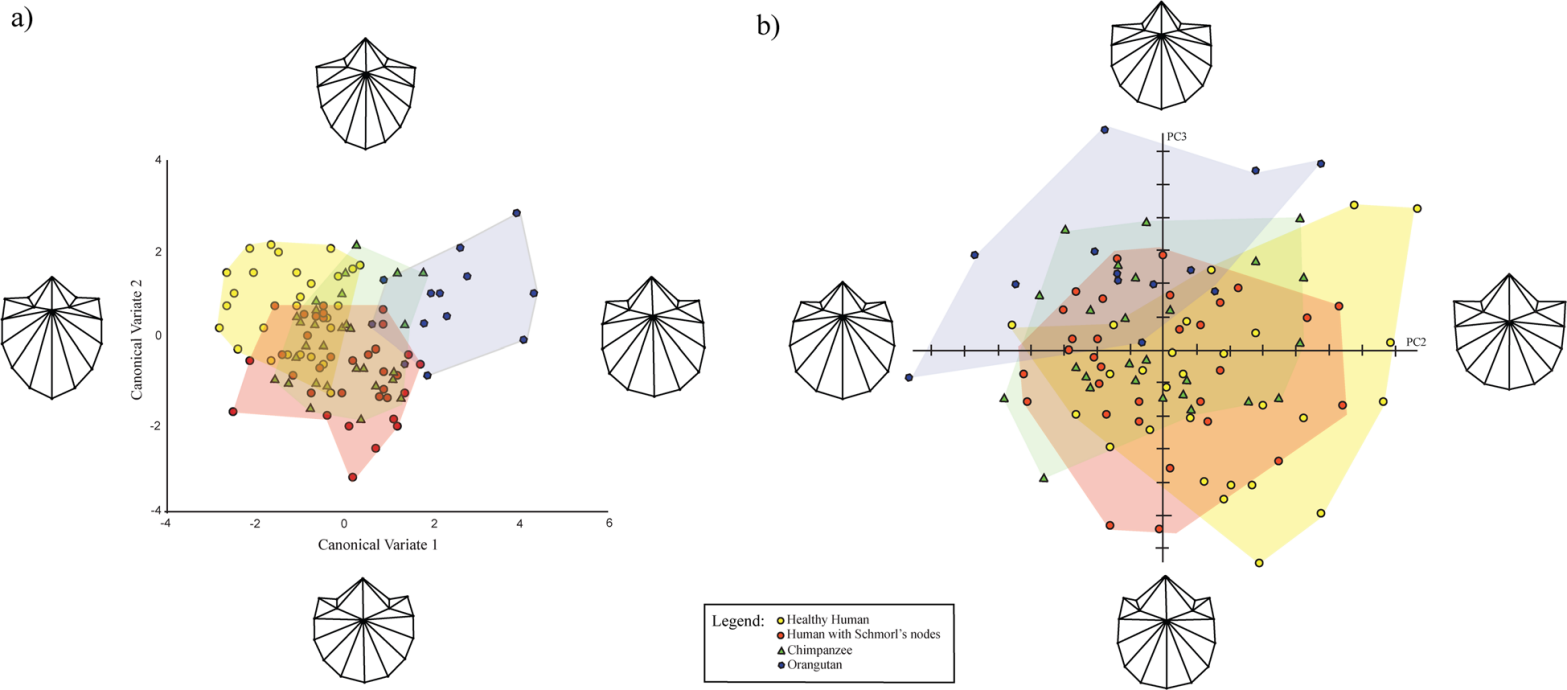
**CVA and PCA plots depicting shape variance of T12/T13 vertebrae. a)** CVA scatter-plot illustrating shape variation of healthy human, pathological human, *P. troglodytes*, *P. pygmaeus* vertebrae on CV1 and CV2 for T12/T13 vertebrae **b)** PCA scatter-plot illustrating shape variance of healthy human, pathological human, *P. troglodytes*, *P. pygmaeus* vertebrae on PC2 and PC3 of T12/T13 vertebrae. Deformation grids illustrate shape differences occurring on each PC.

The CVA of the Procrustes coordinates for the last thoracic vertebrae returned three CVs (canonical vectors). The first accounts for 67.1% of the variance, the second 24.1%, and the third 8.8%. There is little separation among taxa when CV3 is plotted against CV1 (Additional file 2: Figure S1). When CV1 is plotted against CV2 (Figure 2a), it is apparent that the shape of the last thoracic vertebrae of orangutans is different from the shape of the last thoracic vertebrae of not only healthy and pathological humans but also of chimpanzees. It is also apparent when CV1 is plotted against CV2, that pathological human vertebrae have more in common with chimpanzee vertebrae than do healthy humans. All the inter-taxon Procrustes distances are significant except for the one between pathological humans and chimpanzees (Table 2). Pair-wise analyses using permutations of Mahalanobis distances produce the same pattern (Additional file 3: Table S1).

**Table 2:** **Procrustes distances between taxon means for T12/T13 vertebra shape**

	**Pathological humans**	**Orangutans**	**Chimpanzees**
**Healthy humans**	0.0248	0.0539	0.0248
	p = 0.018*	p < 0.0001*	p = 0.028*
**Chimpanzees**	0.0119	0.0352	
	p = 0.5190	p = 0.012*	
**Orangutans**	0.0402		
	p < 0.0001*		

*indicates significant value.

The PCA yielded six PCs that met the ≥5% of variance criterion. PC1 accounts for 30.3% of the variance, PC2 25.6%, PC3 18.1%, PC4 9.6%, PC5 5.1%, and PC6 4.8%. There is considerable overlap among the taxa on PC1, PC4, PC5, and PC6 (Additional file 2: Figure S2-S4). However, the taxa are distinguishable when PC2 and PC3 are plotted against each other. Healthy human vertebrae tend to score more positively on PC2 and negatively on PC3, while orangutan vertebrae tend to score more negatively on PC2 and more positively on PC3 (Figure 2b). Pathological humans and chimpanzees plot between healthy humans and orangutans on both PCs. The deformation grids in Figure 2b illustrate the shape differences between the negative and positive extremes of PC2 and PC3. Moving from the positive extreme of PC2 to the negative one, there is a transition from heart-shaped vertebral bodies with flared pedicles to rounder vertebral bodies without flared pedicles. There is also a decrease in neural foramen size relative to the vertebral body, and a translation of the posterior margin of the body into the neural canal. The shape differences that occur as we move from negative to positive scores on PC3 are a relative decrease in neural foramen size and a relative increase in the width of the pedicles. Thus, compared to healthy humans, pathological humans and chimpanzees have relatively smaller neural foramina, shorter, wider pedicles, and rounder vertebral bodies, whereas compared to orangutans, they have relatively larger neural foramina, longer, narrower pedicles, and more heart-shaped vertebral bodies. The MANOVA on the PCs that met the criterion for inclusion is significant (p < 0.0001). Pair-wise MANOVAs are significant for all inter-taxon comparisons, except those between pathological humans and chimpanzees (Table 3).

**Table 3:** **Results of pairwise MANOVAs for T12/T13 vertebrae on PCs 1 through 6, which collectively represent 93.5% of the total shape variance**

	**Pathological humans**	**Orangutans**	**Chimpanzees**
**Healthy humans**	λ 0.745 F = 3.762 p = 0.005*	λ 0.374 F = 11.362 p < 0.0001*	λ 0.728 F = 3.804 p = 0.005*
**Chimpanzees**	λ 0.986 F = 0.164 p = 0.975	λ 0.537 F = 6.377 p < 0.0001*	
**Orangutans**	λ 0.668 F = 4.077 p < 0.004*		

*indicates significant value.

Thus, the results of the analyses of the last thoracic vertebrae are consistent with the test predictions. The finding of differences among healthy human, chimpanzee, and orangutan vertebrae is in line with the prediction that the vertebral shape of these taxa should be distinguishable due to their locomotion. The analyses also indicate that healthy human vertebrae are statistically distinguishable from chimpanzee vertebrae, whereas pathological human vertebrae are not. This finding is consistent with the prediction that human vertebrae with evidence of vertical intervertebral disc herniation should be more similar in shape to the vertebrae of chimpanzees than are human vertebrae without evidence of intervertebral disc herniation.

### First lumbar vertebrae

**Figure 3:**
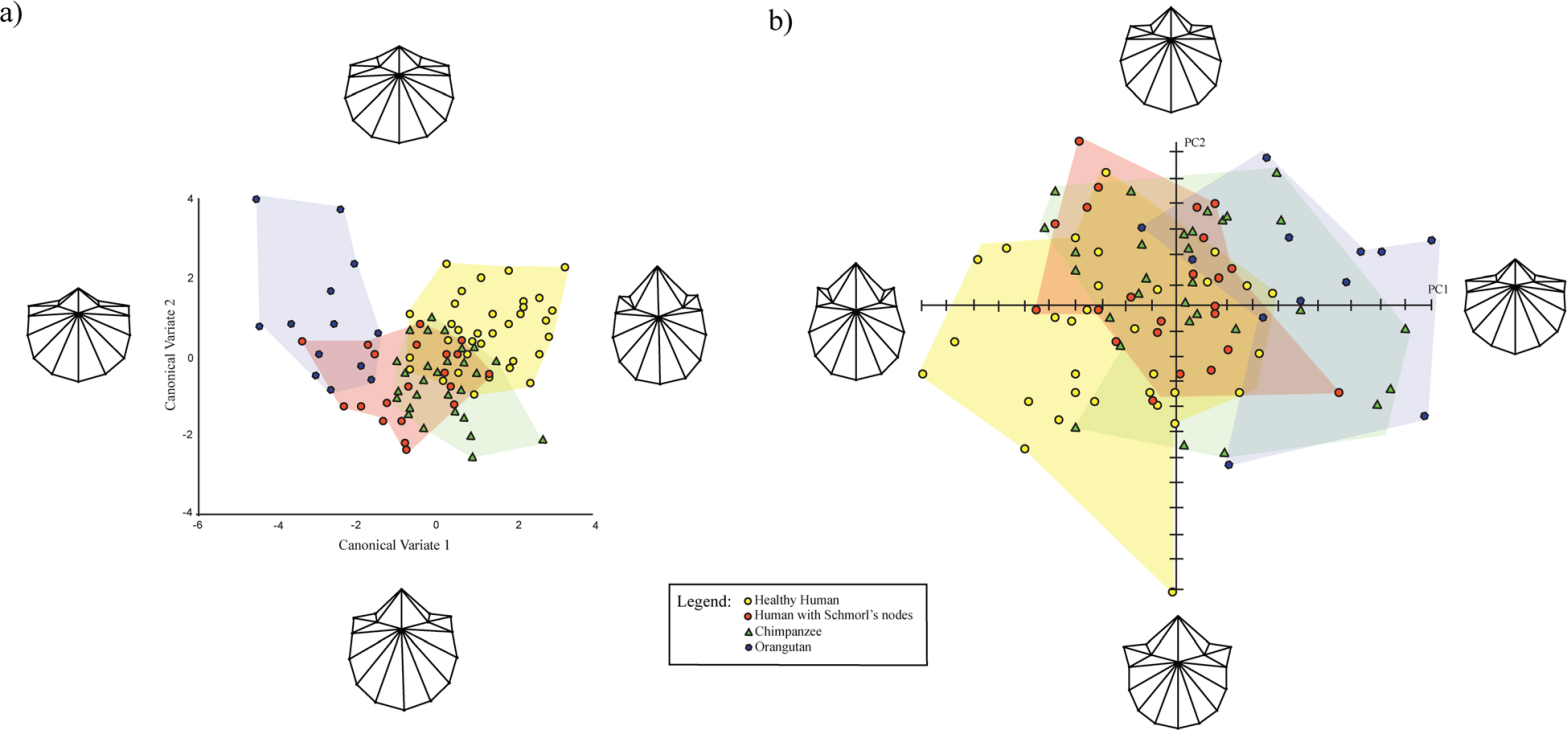
**CVA and PCA plots depicting shape variance of L1 vertebrae. a)** CVA scatter-plot illustrating shape variation of healthy human, pathological human, *P. troglodytes*, *P. pygmaeus* vertebrae on CV1 and CV2 for L1 vertebrae **b)** PCA scatter-plot illustrating shape variance of healthy human, pathological human, *P. troglodytes*, *P. pygmaeus* vertebrae on PC1 and PC2 of L1 vertebrae. Deformation grids illustrate shape differences occurring on each PC.

The CVA of the Procrustes coordinates for the first lumbar vertebrae returned three CVs. The first CV accounts for 68.6% of the variance, the second 20.1%, and the third 11.3%. There is little distinction among the taxa when CV3 is plotted against CV1 (Additional file 2: Figure S5). In contrast, when CV2 is plotted against CV1, it is apparent that pathological human vertebrae are more similar in shape to chimpanzee vertebrae than are healthy human vertebrae (Figure 3a). The Procrustes distances support these observations. All inter-taxon Procrustes distances are significant except the one between pathological human and chimpanzee vertebrae (Table 4). The same pattern is produced by pair-wise analyses using permutations of Mahalanobis distances (Additional file 3: Table S2).

**Table 4:** **Procrustes distances between taxon means for first lumbar vertebra shape**

	**Pathological humans**	**Orangutans**	**Chimpanzees**
**Healthy humans**	0.0303	0.0779	0.0367
	p = 0.004*	p < 0.0001*	p = 0.0004*
**Chimpanzees**	0.0161	0.0458	
	p = 0.4590	p = 0.0001*	
**Orangutans**	0.0549		
	p < 0.0001*		

*indicates significant value.

The PCA for the first lumbar vertebrae yielded five PCs that met the ≥5% of variance criterion. PC1 accounts for 39.6% of the variance, PC2 23.8%, PC3 16.0%, PC4 7.4%, and PC5 5.1%. There is considerable overlap among taxa on PCs 3 through 5 (Additional file 2: Figure S6-S7). However, taxa are distinguishable on PC1 and PC2 (Figure 3b). Healthy humans score more negatively than orangutans on PC1 and PC2, with pathological humans and chimpanzees between them on both PCs. Deformation grids show that the shape differences between samples are similar to those seen in the T12/13 analysis (Figure 3b). Again, the most obvious shape differences relate to the pedicles and vertebral body. Moving from the negative end of PC1 to the positive end, there is a decrease in neural foramen size relative to the vertebral body and the pedicles become shorter and wider. In addition, there is a backward translation of the posterior margin of the vertebral body that results in it becoming less heart-shaped and more shovel-shaped. The shape differences captured by PC2 are a difference in pedicle orientation, with the pedicles becoming more laterally angled from the body as we move from the positive end of PC2 to the negative one. To reiterate, healthy humans and orangutans score at the extremes of the shape variation on both PCs, with pathological humans and chimpanzees between them. Thus, when compared to healthy humans, pathological humans and chimpanzees tend to have smaller neural foramina, wider, shorter pedicles, and more shovel-shaped bodies. When compared to orangutans, pathological humans and chimpanzees have larger neural foramina, narrow pedicles, and less shovel-shaped vertebral bodies. The MANOVA on the PCs that met the ≥5% of variance criterion is statistically significant (p = 0.001). Pair-wise MANOVAs are significant for all inter-taxon comparisons, except between pathological humans and chimpanzees (Table 5).

**Table 5:** **Results of pairwise MANOVAs for first lumbar vertebra on PCs 1 through 5, which collectively represent 92.0% of the total shape variance**

	**Pathological humans**	**Orangutans**	**Chimpanzees**
**Healthy humans**	λ 0.781 F = 2.744 p = 0.029*	λ 0.409 F = 11.854 p < 0.0001*	λ 0.723 F = 4.513 p = 0.002*
**Chimpanzees**	λ 0.892 F = 1.113 p = 0.367	λ 0.640 F = 4.277 p = 0.003*	
**Orangutans**	λ 0.445 F = 6.985 p < 0.0001*		

*indicates significant value.

Thus, the results of the analyses of the first lumbar vertebrae are also consistent with the test predictions. The finding of differences in shape between the healthy human, chimpanzee, and orangutan specimens is consistent with the first test prediction, while the finding that pathological human vertebrae are closer in shape to chimpanzees than are healthy human vertebrae is consistent with the second test prediction.

## Discussion

This study explicitly tested the ancestral shape hypothesis, which holds that intervertebral disc herniation preferentially affects individuals with vertebrae that are towards the ancestral end of the range of shape variation within *H. sapiens* and therefore are less well adapted for bipedalism. We tested two predictions of this hypothesis with shape data recorded on the last thoracic and first lumbar vertebrae of orangutans, chimpanzees, healthy humans, and humans with Schmorl’s nodes, which are bony indicators of intervertebral disc herniation. The first prediction was that there should be differences in shape between healthy human vertebrae, chimpanzee vertebrae, and orangutan vertebrae, due to the different modes of locomotion of the taxa. The second prediction was that pathological human vertebrae should share more similarities in shape with chimpanzee or orangutan vertebrae than do healthy human vertebrae. The results of the analyses were consistent with both predictions. We found that the last thoracic and first lumbar vertebrae of healthy humans, orangutans, and chimpanzees differ significantly in shape, which is in line with the first prediction. We also found that human vertebrae with Schmorl’s nodes share more similarities in shape with chimpanzee vertebrae than do healthy human vertebrae, which is consistent with the second prediction. Thus, the study supports the ancestral shape hypothesis.

A potential alternative explanation for our findings needs to be considered. The vertebral shapes associated with Schmorl’s nodes may be a consequence of intervertebral disc herniation rather than its cause. It is certainly the case that vertebrae can remodel. For example, the shape of the vertebral body is known to change with increasing age. Body height tends to decrease and there is often an increase in surface concavity as the endplate collapses [59]. However, we do not consider intervertebral disc herniation causing changes in vertebral shape to be a good explanation for our results. One of the main shape differences identified between healthy human vertebrae and those with Schmorl’s nodes relates to the neural foramen [25]. Previous work indicates that the shape of the neural foramen does not change after the neural arch fuses to the vertebral body [60,61] at around six years of age in humans [62]. Therefore, any factor that influences the shape of the neural foramen must act during spinal development. Bone remodelling during development could influence the shape of the vertebrae, including the neural foramen. Although this could explain why there is a difference in shape between pathological and healthy human vertebrae, it does not explain the relationship identified between pathological human and chimpanzee vertebrae. This explanation would require that bone remodelling result in vertebral shape changes that systematically approach a shape functionally related to quadrupedal locomotion. This, we submit, is less parsimonious than the ancestral vertebral hypothesis.

A possible functional explanation for the association between vertical disc herniation and vertebral shape is provided by Harrington et al. [24]. These authors suggest that the diameter of the vertebral disc influences its ability to withstand tension during compression. Their argument rests on LaPlace’s law [62], which states that the ability of a fluid-filled tube to withstand tension decreases with increasing radius. According to Harrington et al. [24], the rounder bodies of pathological vertebrae would have a larger diameter than the more heart-shaped bodies seen in healthy vertebrae, making the intervertebral disc less able to withstand stress [24,62]. We also found that pathological vertebrae have shorter pedicles compared to healthy vertebrae. The pedicles act as structural buttresses for the vertebral body and play an important role in load bearing during axial compression [63-68]. It has been hypothesized that the shorter pedicles identified in vertebrae with Schmorl’s nodes may be less able to withstand physical strain placed on the spine [25,45]. Since bipedalism causes a large amount of axial loading on the lower vertebrae [30], we hypothesize that the combination of round vertebral bodies with short pedicles may provide less support for the spine during bipedal posture and locomotion.

Our results have implications for medical science beyond shedding light on the causes of intervertebral disc herniation. One is that vertebral shape may be a factor that could help predict an individual’s susceptibility to vertical intervertebral disc herniation. The shape analysis techniques used in this study can also be used on medical images, such as CT scans. It may be possible for clinicians to investigate an individual’s vertebral shape and identify those who may be at risk of developing the condition. This ability would have significant diagnostic and preventative value, especially for high-risk individuals, such as athletes [69]. In addition, a better understanding of the role that locomotion and posture plays in the health of the spine could aid in the treatment of individuals afflicted with symptomatic vertical intervertebral disc herniation. Locomotion is recognized as an important factor in rehabilitation for sufferers of back pain [70], and understanding the role that vertebral variation can play in spinal health could aid physiotherapists to refine activity and exercise regimes. Thus, the findings of this study may not only help medical practitioners to understand why some individuals are more commonly afflicted with back problems than others, but may also lead to advances in the identification, prevention, and treatment of people suffering from intervertebral disc herniation.

In addition to offering these potential clinical benefits, our results provide further support for the claim that an evolutionary perspective can shed important light on human health problems [71-74]. Evolutionary medicine has identified the value of considering evolutionary adaptations to enable better understanding of human developmental issues, chronic diseases, and nutritional needs [74], but the influence of skeletal morphology on human health has received little attention. Our study highlights the potential of using osteological analyses of skeletal variation, including comparative analyses between humans and non-human primate species, in evolutionary medical studies. Bipedalism has been suggested to impact human spinal and joint health [28-30,75,76], but few studies have been carried out to evaluate this proposition [30]. The identification of an ancestral vertebral shape that influences the occurrence of a common spinal pathology supports the idea that the relatively rapid evolution of bipedalism in the hominins may continue to impact modern human health.

The main goal of our study was to shed light on a major contemporary health problem with the conceptual and analytical tools of evolutionary biology, but our results also contribute to the understanding of human evolution. Specifically, they shed additional light on the evolution of bipedalism, and in particular, the functional anatomy associated with it. Previous studies have identified morphological characteristics purported to relate to bipedalism [77-80]. The present findings add features to this list—a larger neural foramen relative to body size, taller, narrower pedicles, and a more heart-shaped vertebral body. There are two persistent debates in palaeoanthropology regarding the evolution of bipedalism and a better understanding of the functional anatomy of bipedal vertebrae may contribute to their resolution. The first debate regards the timing of the emergence of bipedalism in the evolutionary record. The understanding of how human vertebrae are unique among hominoids enables the identification of fossil vertebrae adapted for bipedal locomotion; this will help researchers infer which species were bipedal, provide additional insight into how bipedalism evolved, and suggest whether it followed a gradual or punctuated pattern of evolution. The second debate surrounding the evolution of bipedalism is whether early bipeds walked with their knees and hips in a flexed position, like chimpanzees, or if their mode of bipedalism resembled our own [81-84]. The ability to identify vertebral shape characteristics unique to humans and compare these with features unique to modern chimpanzees may provide additional insight into the functional anatomy required for habitual bipedalism and help understand the evolutionary trends that led to the modern human gait.

With regard to future research, several possibilities suggest themselves. Firstly, if the ancestral shape hypothesis is accepted, it prompts the question of how this shape influences the occurrence of vertical intervertebral disc herniation. This could be investigated with biomechanical studies of the interaction between locomotion, vertebral morphology, and the soft tissues of the spine. Secondly, this area of research would benefit from the use of 3D shape analyses of human and non-human ape vertebrae to investigate how 3D vertebral morphology relates to locomotion and human spinal health. Lastly, the clinical value of this research would be substantially increased with the inclusion of in-vivo medical images of individuals with and without back problems.

## Conclusions

Our study supports the hypothesis that intervertebral disc herniation preferentially affects individuals with vertebrae that are towards the ancestral end of the range of shape variation within *Homo sapiens* and therefore are less well adapted for bipedalism. As predicted by the hypothesis, we identified a relationship between the shape of the last thoracic and first lumbar vertebrae and locomotion in humans, chimpanzees, and orangutans, and we found that human vertebrae with signs of vertical intervertebral disc herniation are indistinguishable from those of chimpanzees. When compared to healthy humans, pathological human and chimpanzee vertebrae tend to have smaller neural foramina, shorter, wider pedicles, and more shovel-shaped vertebral bodies. Our study’s support for the ancestral shape hypothesis not only has clinical implications, but also provides another illustration of the benefits of bringing the conceptual and analytical tools of evolutionary biology to bear on problems in medicine and public health.

## Additional files

Additional file 1:
**Archaeological site information for Fishergate House, York, and Coach Lane, North Shields, Tyne and Wear.**
Additional file 2: Figure S1. CVA scatter-plot illustrating shape variance of healthy human, pathological humans, *P. troglodytes*, *P. pygmaeus* vertebrae on CV1 and CV3 for T12/T13 vertebrae. **Figure S2.** PCA scatter-plot illustrating shape variance on PC1 and PC2 for T12/T13 vertebrae. Legend: yellow circle - healthy humans, red circles – pathological humans, green triangles – chimpanzees, blue octagons – orangutans. **Figure S3.** PCA scatter-plot illustrating shape variance on PC4 and PC5 for T12/T13 vertebrae. Legend: yellow circle - healthy humans, red circles – pathological humans, green triangles – chimpanzees, blue octagons – orangutans. **Figure S4.** PCA scatter-plot illustrating shape variance on PC5 and PC6 for T12/T13 vertebrae. Legend: yellow circle - healthy humans, red circles – pathological humans, green triangles – chimpanzees, blue octagons – orangutans. **Figure S5.** CVA scatter-plot illustrating shape variance of healthy human, pathological humans, *P. troglodytes*, *P. pygmaeus* vertebrae on CV1 and CV3 for L1 vertebrae. **Figure S6.** PCA scatter-plot illustrating shape variance on PC2 and PC3 for L1 vertebrae. Legend: yellow circle - healthy humans, red circles – pathological humans, green triangles – chimpanzees, blue octagons – orangutans. **Figure S7.** PCA scatter-plot illustrating shape variance on PC4 and PC5 for L1 vertebrae. Legend: yellow circle - healthy humans, red circles – pathological humans, green triangles – chimpanzees, blue octagons – orangutans. Additional file 3: Table S1. Mahalanobis distances between taxon means for the last thoracic vertebrae shape. **Table S2.** Mahalanobis distances between taxon means for first lumbar vertebrae shape.
