# Supplementary material for: The ancestral shape hypothesis: an evolutionary explanation for the occurrence of intervertebral disc herniation in humans
Source: BMC Evol Biol. 2015 Apr 27;15:68. doi: 10.1186/s12862-015-0336-y (PMC4410577; doi:10.1186/s12862-015-0336-y)
Supplement: Additional file 1: — Archaeological site information for Fishergate House, York, and Coach Lane, North Shields, Tyne and Wear. [file 12862_2015_336_MOESM1_ESM.pdf]

Additional File 1

Supplementary Information

Archaeological site information:

Fishergate House, York:

This site was excavated by Field Archaeology Specialists Limited in association with Mike Griffiths Associates on behalf of Shepherd Homes and Rank leisure from 2000 to 2002. The skeletal report was completed by Malin Holst of York Osteoarchaeology Limited (Holst 2005). There were 244 skeletons in a general good state of preservation, including 52 definite females and 49 definite male. The cemetery burials were dated to the late medieval period. The details of this cemetery remain unclear, however, the individuals were likely that of the local lay population in York (Holst 2005)(https://www.dur.ac.uk/archaeology/facilities_services/fhol/).

Coach Lane, North Shields, Tyne and Wear:

The site at Coach Lane, North Sheilds Quaker cemetery called the Society of Friends burial ground which was in use from 1711 to 1857 AD. It was excavated in 2010 by Pre-Construct Archaeology Ltd found 236 skeletons, including 50 females or possible females and 45 males or possible male skeletons (Langthorne, 2012). The skeletal analysis and report was completed by Jenny Proctor and James Langthorne.
