# Supplementary material for: The ancestral shape hypothesis: an evolutionary explanation for the occurrence of intervertebral disc herniation in humans
Source: BMC Evol Biol. 2015 Apr 27;15:68. doi: 10.1186/s12862-015-0336-y (PMC4410577; doi:10.1186/s12862-015-0336-y)
Supplement: Additional file 2: Figure S1. — CVA scatter-plot illustrating shape variance of healthy human, pathological humans, P. troglodytes, P. pygmaeus vertebrae on CV1 and CV3 for T12/T13 vertebrae. Figure S2. PCA scatter-plot illustrating shape variance on PC1 and PC2 for T12/T13 vertebrae. Legend: yellow circle - healthy humans, red circles – pathological humans, green triangles – chimpanzees, blue octagons – orangutans. Figure S3. PCA scatter-plot illustrating shape variance on PC4 and PC5 for T12/T13 vertebrae. Legend: yellow circle - healthy humans, red circles – pathological humans, green triangles – chimpanzees, blue octagons – orangutans. Figure S4. PCA scatter-plot illustrating shape variance on PC5 and PC6 for T12/T13 vertebrae. Legend: yellow circle - healthy humans, red circles – pathological humans, green triangles – chimpanzees, blue octagons – orangutans. Figure S5. CVA scatter-plot illustrating shape variance of healthy human, pathological humans, P. troglodytes, P. pygmaeus vertebrae on CV1 and CV3 for L1 vertebrae. Figure S6. PCA scatter-plot illustrating shape variance on PC2 and PC3 for L1 vertebrae. Legend: yellow circle - healthy humans, red circles – pathological humans, green triangles – chimpanzees, blue octagons – orangutans. Figure S7. PCA scatter-plot illustrating shape variance on PC4 and PC5 for L1 vertebrae. Legend: yellow circle - healthy humans, red circles – pathological humans, green triangles – chimpanzees, blue octagons – orangutans. [file 12862_2015_336_MOESM2_ESM.docx]

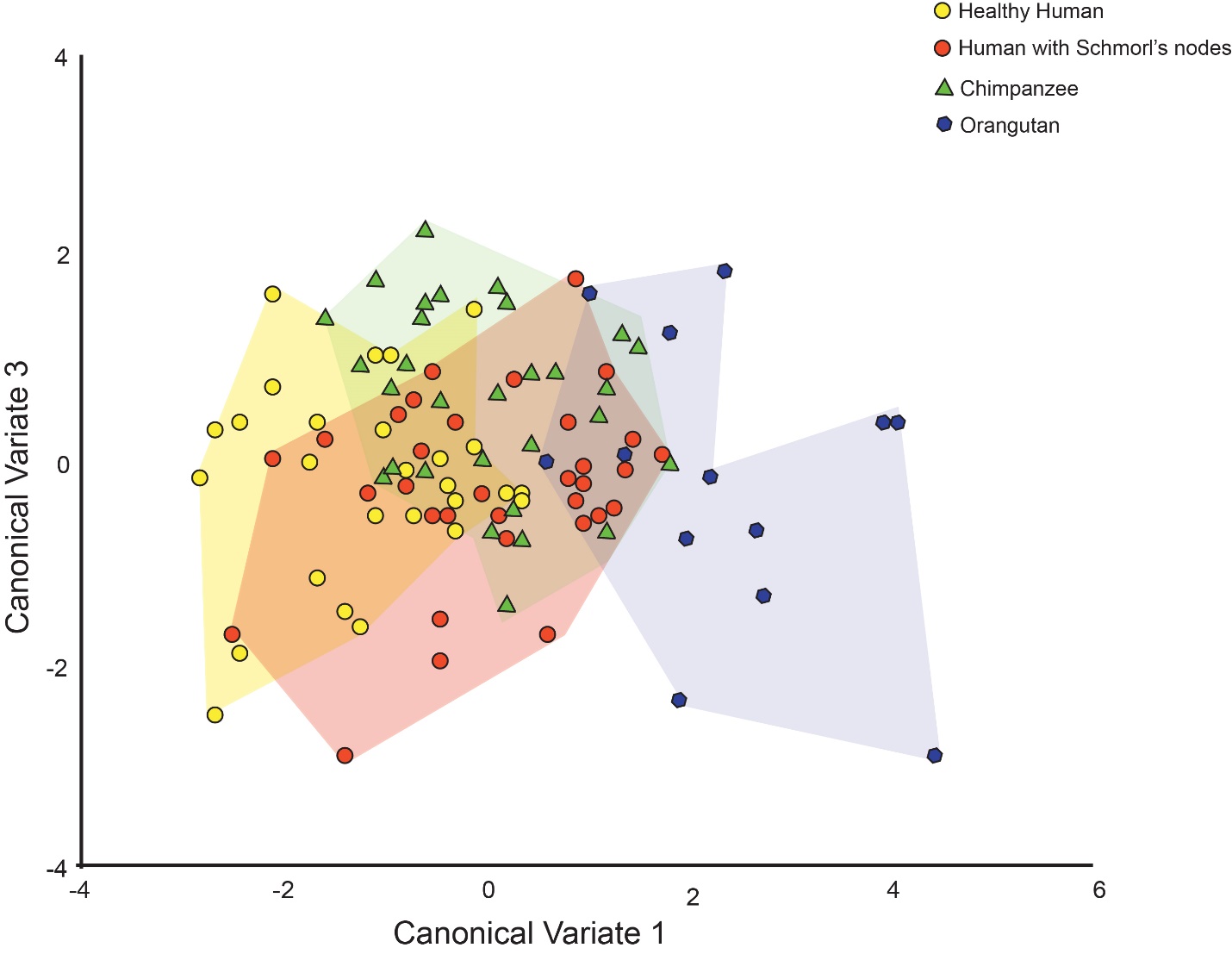


Figure S1) CVA scatter-plot illustrating shape variance of healthy human, pathological humans, *P. troglodytes*, *P. pygmaeus* vertebrae on CV1 and CV3 for T12/T13 vertebrae.


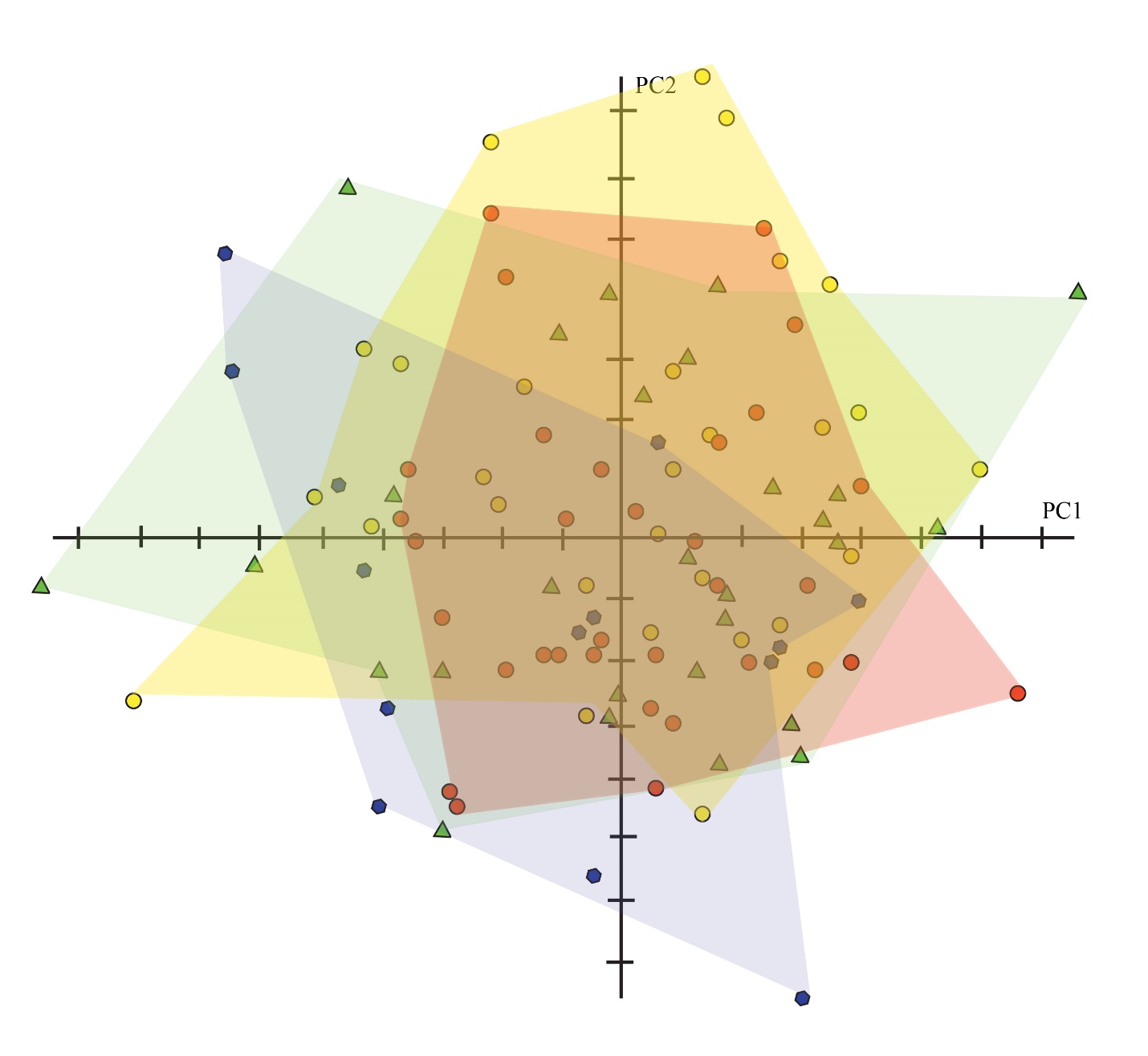


Figure S2) PCA scatter-plot illustrating shape variance on PC1 and PC2 for T12/T13 vertebrae. Legend: yellow circle - healthy humans, red circles – pathological humans, green triangles – chimpanzees, blue octagons – orangutans.


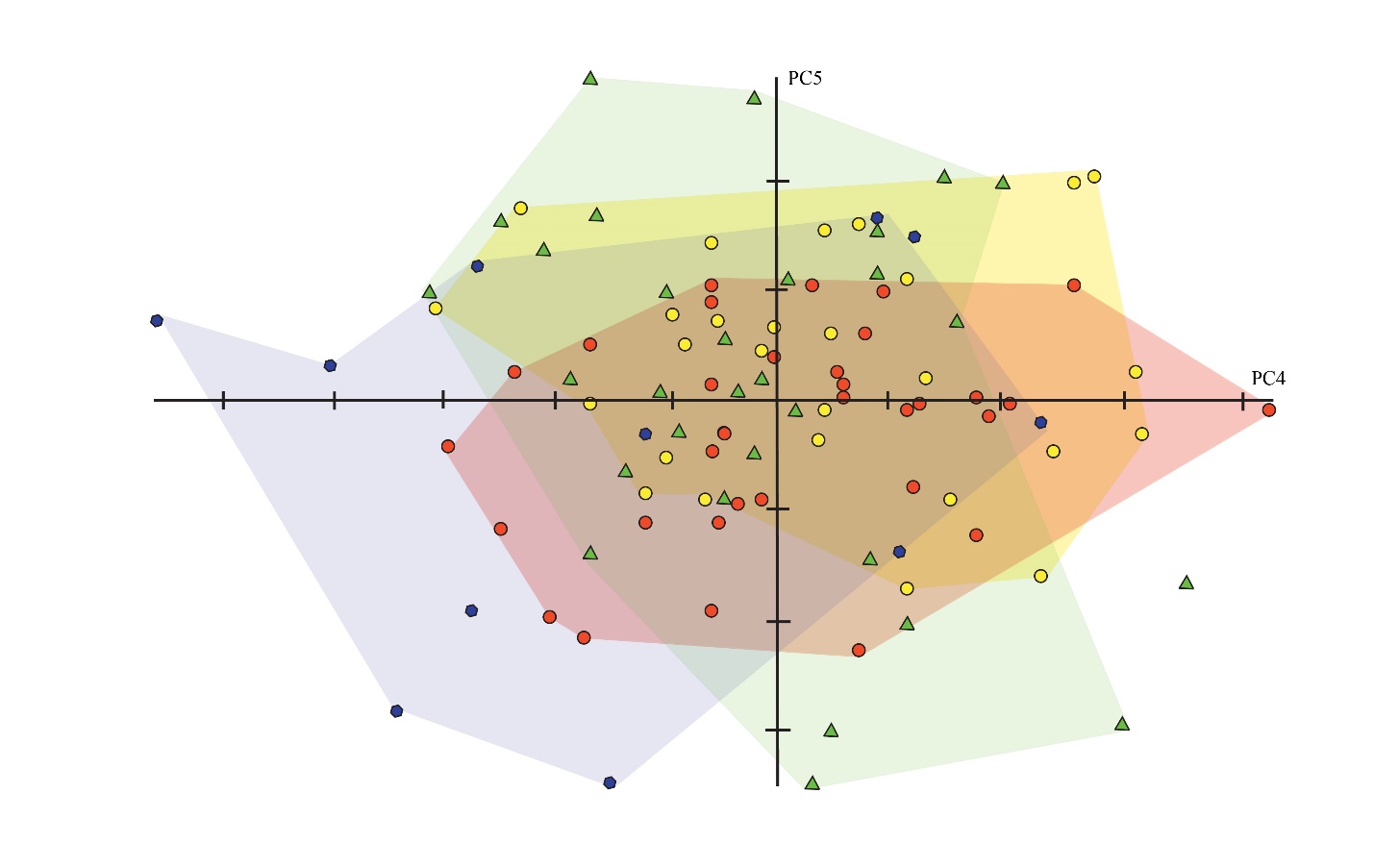


Figure S3) PCA scatter-plot illustrating shape variance on PC4 and PC5 for T12/T13 vertebrae. Legend: yellow circle - healthy humans, red circles – pathological humans, green triangles – chimpanzees, blue octagons – orangutans.


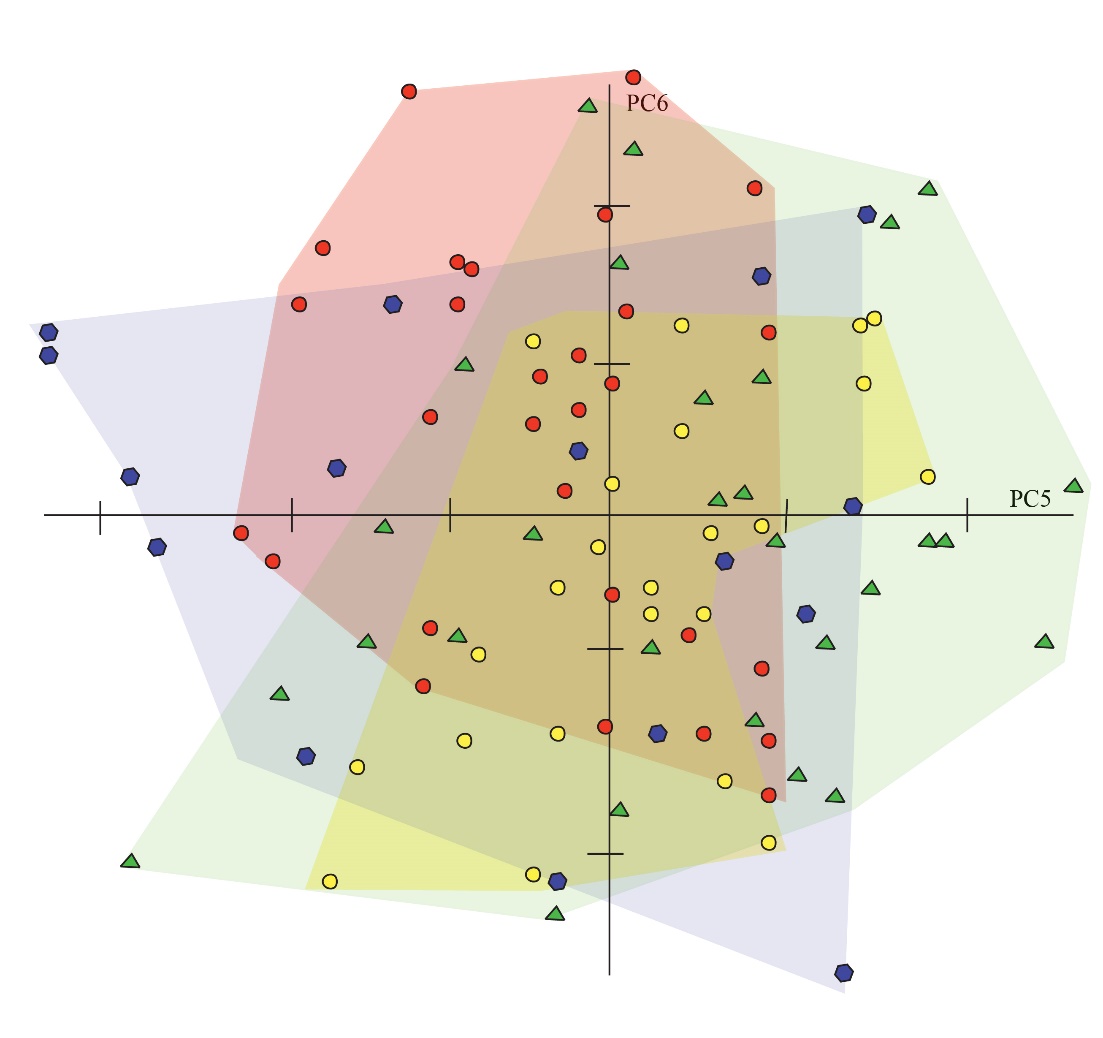


Figure S4) PCA scatter-plot illustrating shape variance on PC5 and PC6 for T12/T13 vertebrae. Legend: yellow circle - healthy humans, red circles – pathological humans, green triangles – chimpanzees, blue octagons – orangutans.


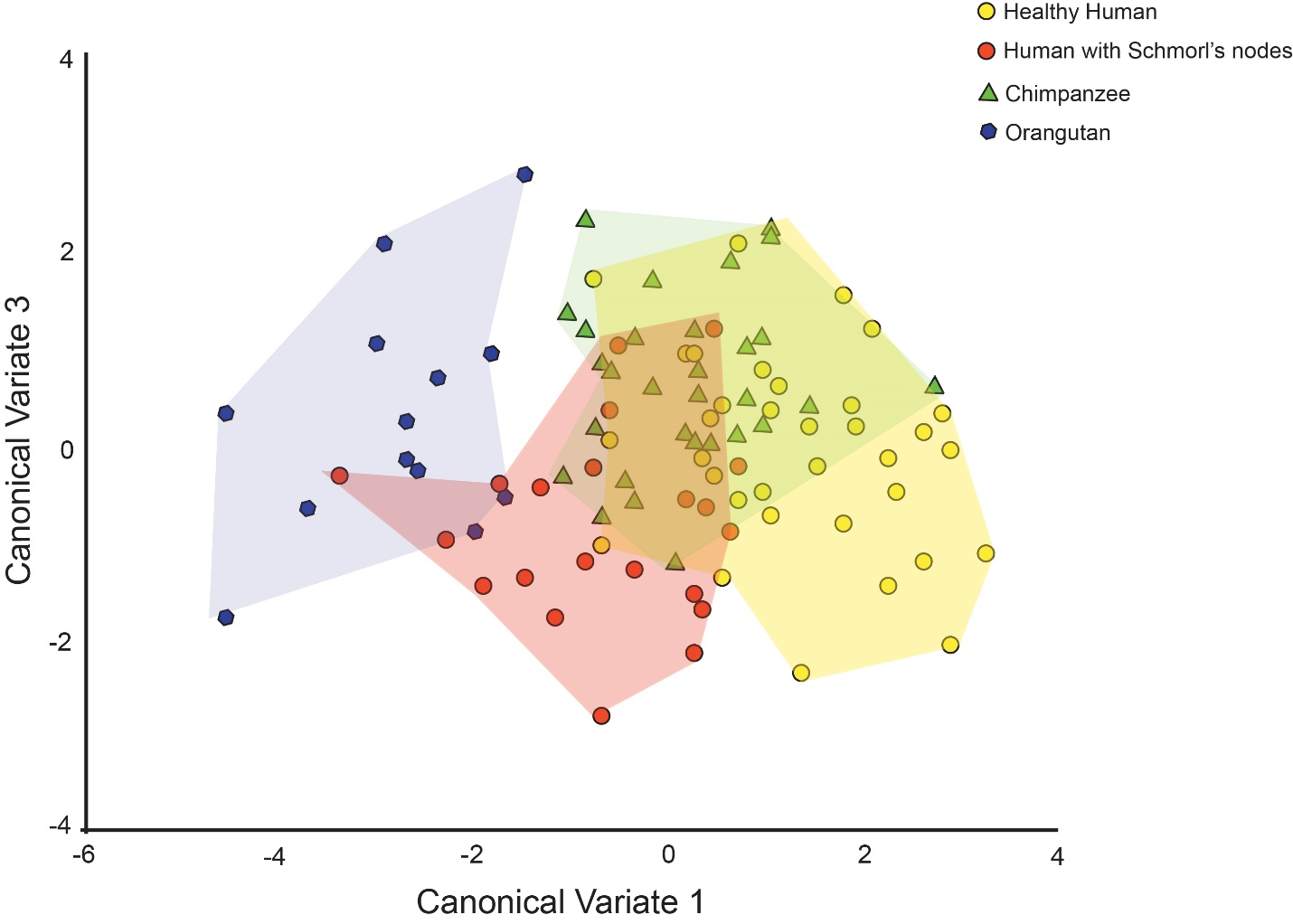


Figure S5) CVA scatter-plot illustrating shape variance of healthy human, pathological humans, *P. troglodytes*, *P. pygmaeus* vertebrae on CV1 and CV3 for L1 vertebrae.


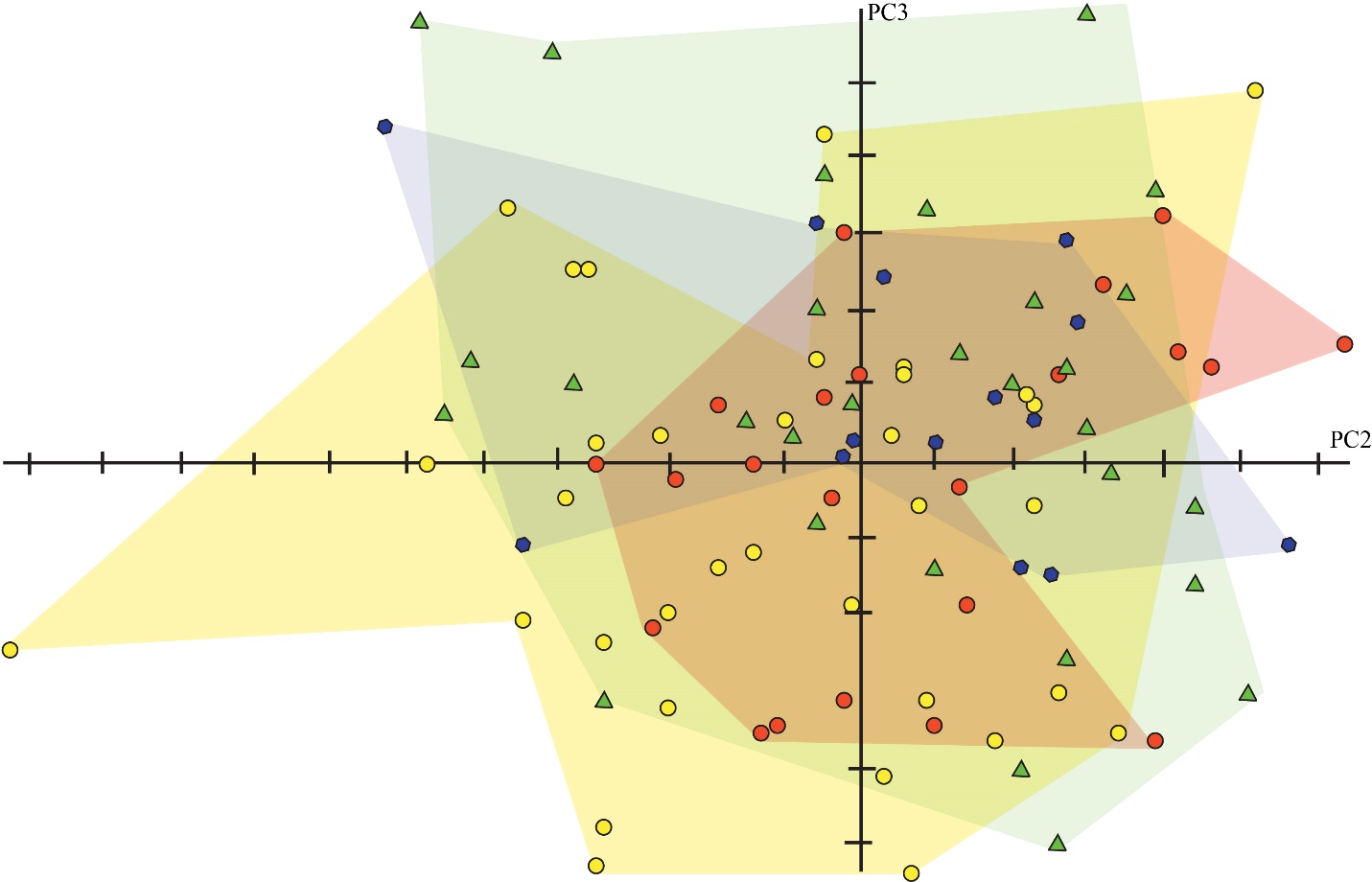


Figure S6) PCA scatter-plot illustrating shape variance on PC2 and PC3 for L1 vertebrae. Legend: yellow circle - healthy humans, red circles – pathological humans, green triangles – chimpanzees, blue octagons – orangutans.


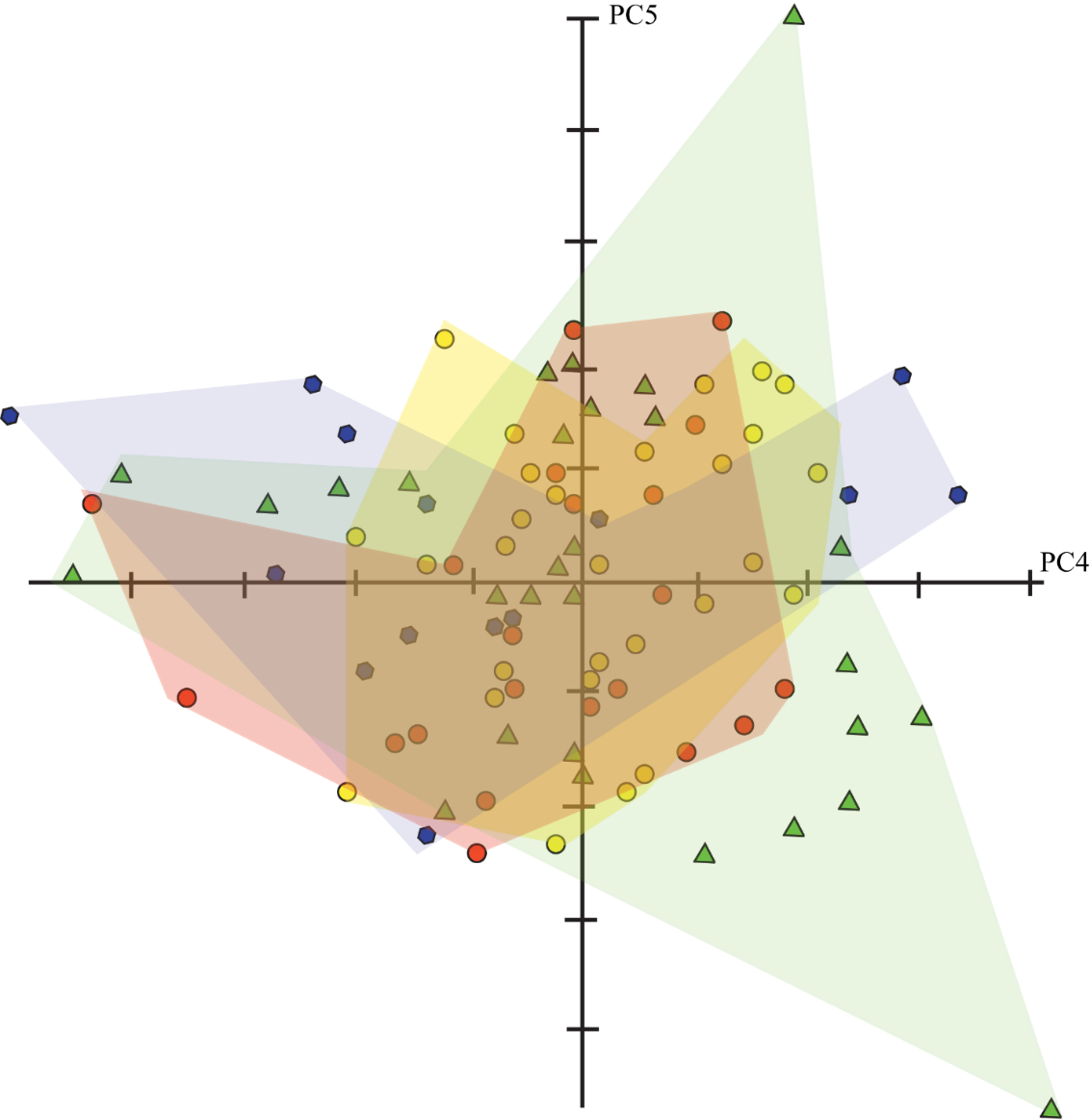


Figure S7) PCA scatter-plot illustrating shape variance on PC4 and PC5 for L1 vertebrae. Legend: yellow circle - healthy humans, red circles – pathological humans, green triangles – chimpanzees, blue octagons – orangutans.
