# Supplementary material for: The ancestral shape hypothesis: an evolutionary explanation for the occurrence of intervertebral disc herniation in humans
Source: BMC Evol Biol. 2015 Apr 27;15:68. doi: 10.1186/s12862-015-0336-y (PMC4410577; doi:10.1186/s12862-015-0336-y)
Supplement: Additional file 3: Table S1. — Mahalanobis distances between taxon means for the last thoracic vertebrae shape. Table S2. Mahalanobis distances between taxon means for first lumbar vertebrae shape. [file 12862_2015_336_MOESM3_ESM.pdf]

Table S1. Mahalanobis distances between taxon means for the last thoracic vertebrae shape.

	**Pathological humans**	**Orangutans**	**Chimpanzees**
**Healthy humans**	2.0798 p<0.0001*	3.5703 p<0.0001*	1.7574 p=0.006*
**Chimpanzees**	1.0980 p=0.9490	2.7921 p<0.0001*	
**Orangutans**	2.8741 p<0.0001*		

Table S2. Mahalanobis distances between taxon means for first lumbar vertebrae shape.

	**Pathological humans**	**Orangutans**	**Chimpanzees**
**Healthy humans**	2.7884 p<0.0001*	3.9399 p<0.0001*	2.0661 p<0.0001*
**Chimpanzees**	1.7036 p<0.1429	2.9034 p<0.0001*	
**Orangutans**	2.6087 p=0.0002*		
